# Fibrosis in Mesothelioma: Potential Role of Lysyl Oxidases

**DOI:** 10.3390/cancers14040981

**Published:** 2022-02-15

**Authors:** Lara Perryman, Steven G. Gray

**Affiliations:** 1 Drug Discovery Department, Pharmaxis Ltd., Sydney, NSW 2086, Australia; lara.perryman@pharmaxis.com.au; 2 Thoracic Oncology, Labmed Directorate, St James’s Hospital, D08 RX0X Dublin, Ireland

**Keywords:** malignant pleural mesothelioma, fibrosis, collagen, lysyl oxidase, therapy, extracellular matrix, biomarker, stroma

## Abstract

**Simple Summary:**

Why do most drugs have limited effects in difficult-to-treat cancers such as mesothelioma? One explanation commonly given in response is that not enough drug can get into the tumor to generate an anti-tumor effect. Fibrosis is a common element of mesothelioma that causes the area around the cancer to stiffen. By itself, fibrosis makes breathing difficult and causes a poor quality of life, but it also acts as a barrier stopping drugs from getting into the tumor, and limits the immune system’s ability to detect and access the tumor to kill it. In this review, we discuss how a family of proteins called lysyl oxidases is associated with fibrosis in many disease settings, including solid tumors and mesothelioma, the current status of efforts to therapeutically target these proteins and how targeting this family may have therapeutic applicability in the treatment and management of mesothelioma.

**Abstract:**

Immunotherapies (such as checkpoint inhibitors) and standard chemotherapies (such as cisplatin) have limitations in the successful treatment of malignant pleural mesothelioma (MPM). Fibrosis is the accumulation of collagen in the extracellular matrix (ECM) of tissues, making them denser than that of healthy tissues and thereby affecting drug delivery and immune cell infiltration. Moreover, fibrosis severely affects the patient’s breathing and quality of life. The production of collagen and its assembly is highly regulated by various enzymes such as lysyl oxidases. Many solid tumors aberrantly express the family of lysyl oxidases (LOX/LOXL). This review examines how LOX/LOXLs were found to be dysregulated in noncancerous and cancerous settings, discusses their roles in solid tumor fibrosis and pathogenesis and explores the role of fibrosis in the development and poor clinical outcomes of patients with MPM. We examine the current preclinical status of drugs targeting LOX/LOXLs and how the incorporation of such drugs may have therapeutic benefits in the treatment and management of patients with MPM.

## 1. Introduction

Malignant pleural mesothelioma (MPM) is an aggressive inflammatory cancer associated with exposure to asbestos [1]. Industrialized nations’ use of asbestos has declined, but asbestos continues to be exported and used in developing nations. Despite its ban, environmental exposure is still widespread [1,2,3,4]. Early-stage MPM patients typically present with shortness of breath, coughing and chest pains, which is attributed to pleural thickening and pleural effusion. Pleural effusion is an accumulation of fluid in the space between the lungs and the chest wall that can be eased via thoracentesis (aspiration of fluid) or chemical pleurodesis (intrapleural administration of talc obliterating the pleural space). Computed tomography (CT) imaging is routinely performed to detect pleural fluid, present in 90% of MPM patients, although biopsies are required for a definitive diagnosis [5]. Pleural effusion is caused by an imbalance between pleural fluid production and an inadequacy of the lymphatic drainage system, which again limits respiration. As MPM advances, tumor growth further restricts breathing, diminishing quality of life. Untreated, MPM has a median survival time of 8–12 months [6] and a 5-year survival rate from diagnosis of ~7% [7]. For a long time, first-line standard of care (SOC) was a combination of pemetrexed/raltitrexed and cisplatin chemotherapy [8], which results in a response rate of ~40% and is non-curative [9]. Recently, the addition of Bevacizumab to this regimen was approved for first-line therapy [10]. The dual checkpoint inhibitor immunotherapy (Ipilimumab/Nivolumab) was FDA-approved for use in treatment of naive mesothelioma patients [11], based on evidence generated in the CheckMate 743 trial [12]. Despite showing only similar efficacy to chemotherapy for both median progression-free survival (PFS) and objective response rate (ORR), the dual-immunotherapy regimen demonstrated significantly improved duration of response (DOR). Critically, at 24 months, 41% of patients treated with the dual-immunotherapy regimen remained alive as compared with 27% of patients treated with chemotherapy. Dual-immunotherapy may become the new standard of care for MPM [12]. Checkpoint inhibitor trials such as the CONFIRM-1 trial (NCT03063450) suggest potential for patients who have progressed on standard first-line therapy [13]. The cisplatin, pemetrexed and checkpoint inhibitors combination studies, such as the JME-001 (UMIN000030892) and PrE0505 (NCT02899195), in unresectable, malignant pleural mesothelioma are potential first-line therapies [14,15].

Objective response rates for checkpoint inhibitors in MPM, such as in cisplatin/pemetrexed trials, are limited to a proportion (20–30%) of patients [16,17]. There is still an urgent clinical need to identify new translational targets or novel therapeutic approaches to improve patient outcomes. Multi-tyrosine kinases inhibitors may have potential as a therapeutic intervention in MPM [18]. Other examples of potential novel treatments currently in clinical trials include: BRCA1 positivity for sensitivity to vinorelbine (Clinical Trials.gov NCT02139904) [19,20], poly (ADP-ribose) polymerase (PARP) inhibitors (NCT03654833) [20], inhibitors of CDK4/6 (e.g., NCT03654833), inhibitors of EZH2 (NCT02860286) [1], or arginine depletion in Argininosuccinate Synthetase 1-Deficient tumors (NCT02709512, NCT02709512) [21,22].

The deposition of ECM is vital for tissue repair and is reversible in healthy tissue. Excessive accumulation of ECM proteins, such as collagen and fibronectin, leads to organ malfunction and is called fibrosis. The pleural thickening in MPM patients is thought to contain fibrotic tissue, thereby increasing tissue density and stiffness compared to healthy tissues. These ECM alterations potentially affect drug delivery, immune cell infiltration and lymphatic drainage causing pleural effusion, ultimately leading to a poor quality of life for patients [23]. 

The production of collagen and its assembly is highly regulated by various enzymes such as lysyl oxidases. It is now well established that many solid tumors aberrantly express the family of lysyl oxidases (LOX/LOXLs) [23]. Lysyl oxidases are copper-dependent amine oxidases that catalyze the enzymatic step in the crosslinking of collagen and elastin [24]. The lysyl oxidase family consists of five paralogues: LOX and LOX-Like 1, 2, 3 and 4 (LOXL1, LOXL2, LOXL3 and LOXL4) [25]. The expression of LOX/LOXL is considered an indicator of fibrosis and correlates to tissue stiffness in both fibrotic diseases and cancer. Upregulation of LOX/LOXL is common in cancers and fibrotic diseases, whereas in rare cancers such as mesothelioma comparatively little is known. Idiopathic Pulmonary Fibrosis (IPF) is an interstitial fibrosis in the lining of the alveoli that develops in response to inflammation and has an upregulation of lysyl oxidases [26]. In mesothelioma, asbestos accumulation in the pleural space induces chronic inflammation, initiating malignant transformation of mesothelial cells and eventually fibrosis [1,3]. MPM forms a sheath-like tumor around the lung, which is histologically distinct from IPF. It is anticipated there are many parallels in the underlying molecular mechanism of all fibrotic diseases, including the upregulation of lysyl oxidases. 

Generally, lysyl oxidases are secreted proteins; however, additional intracellular roles for these enzymes have been identified. Overexpression of LOXL2 is associated with a more aggressive invasive phenotype, which is associated with an epithelial-mesenchymal-transition (EMT) phenotype and drives cancer metastasis [27]. EMT activation can be due to both mechanosensing/mechanotransduction and potentially intracellular mechanisms of LOX/LOXL2. LOXL2 can translocate to the nucleus and stabilize the transcription factor Snail, an integral step in EMT [27,28]. Additional critical roles have been shown for LOX/LOXL in both cancer-associated fibroblasts (CAFs) and fibrocyte-mediated regulation of the tumor microenvironment [29,30,31,32,33]. Lysyl oxidases are important in multiple mechanisms that induce fibrosis and disease progression and hamper response to current therapies. 

## 2. Altered Expression of Lysyl Oxidases in Non-Cancerous Settings

LOX/LOXLs activity and expression is altered in many non-cancerous lung diseases, all of which have a degree of fibrosis, as summarized in Table 1.

### 2.1. Idiopathic Pulmonary Fibrosis (IPF)

IPF is a clinically challenging condition of the lung with significant interstitial fibrosis, is progressive, irreversible and has a poor prognosis with a median survival of 2–4 years [82]. Recently, a significant amount of evidence has linked the immune microenvironment to the development of fibrosis in IPF [83,84,85], but what evidence links lysyl oxidase activity to the fibrotic process? Numerous studies on bleomycin-induced lung fibrosis have confirmed the induction of LOX expression and the amelioration of fibrosis by the pan-lysyl oxidase inhibitor β-aminopropionitrile (BAPN) (Table 1) [35,36,37,86]. Elevated activity of LOX has been confirmed for other models of IPF, for example in paraquat-induced fibrosis [39,40] (Table 1). Elevated levels of LOXL2 also play important roles in IPF with links to disease progression risk [38], increased fibroblast-to-myofibroblast transition (FMT) [41] and increased collagen fibril thickness [43]. Moreover, the nuclear localization of LOXL2 protein has been associated with the progression of lung fibrosis [45] and suggests a dynamic regulatory role (Table 1). In this regard, inhibition of LOXL2 in ex vivo lung explants from patients with IPF was shown to induce collagen turnover [46] (Table 1). LOXL1 and LOXL3 have also been identified in the pathogenesis of IPF [41,43,47] (Table 1).

### 2.2. Other Non-Cancerous Disease States

A significant body of evidence now also links lysyl oxidases to other non-cancerous fibrotic conditions including renal fibrosis, liver fibrosis and sclerosis (Table 1). In kidney fibrosis, LOXL2 appears to be a major factor with potential utility as a candidate biomarker for monitoring kidney fibrosis [50], and is a critical element in tubulointerstitial fibrosis [48] (Table 1). In models of renal fibrosis, therapeutically targeting LOXL2 activity is able to attenuate the main symptoms [49,51].

Within the setting of the liver, fibrosis is correlated with increased LOX levels in both the tissue and serum of patients, and there is a further association with altered collagen in the extracellular space in animal models of hepatic fibrosis [52,53,54,55,64] (Table 1). Elevated LOX levels in patient serum have since been mapped along the liver fibrosis continuum from chronic persistent hepatitis to chronic active hepatitis to cirrhosis [60]. A critical element in the increased LOX activity and fibrosis in liver tissue involves the activation of hepatic stellate cells (HSCs) [56,57,58,59].

In addition to LOX, links between liver fibrosis and LOXLs have also been identified, spanning non-alcoholic fatty liver disease (NAFLD) to cirrhosis [61,62,67,68,74]. Intriguingly, microRNAs (miRNAs) that have been found to target LOX/LOXLs have been identified in liver fibrosis [63,72,73] (Table 1). It is interesting to note that some of the miRNAs identified have both altered expression in and potential therapeutic impact on MPM. The accumulated evidence that LOX/LOXL activity plays important roles in liver fibrosis suggests that targeting these may have potential therapeutic benefit by reducing fibrosis [65,67,68,69,71,74] (Table 1). 

Sclerosis encompasses a diverse number of pathological conditions and fibrosis is associated with both amyotrophic lateral sclerosis (ALS) and systemic sclerosis. LOX expression and activity are increased in both animal models and patient samples of ALS [75,76], and LOX, LOXL2 and LOXL4 are elevated in the fibroblasts and serums of patients with systemic sclerosis [37,77,78,79,80,81] (Table 1).

Other non-cancerous settings for which lysyl oxidases have been identified to play significant roles include cardiac disease [87,88,89,90], complications associated with diabetes (for example diabetic retinopathy) [91,92,93,94], bone remodeling and osteoporosis [95,96].

## 3. Altered Expression of Lysyl Oxidases in Cancerous Settings

Fibrosis is also commonly associated with many solid tumors [97,98]. In the following sections, we summarize the pertinent information linking LOX/LOXL activity to some of the main solid cancers in Table 2.

### 3.1. Breast Cancer

The role of collagen and fibrosis in breast cancer is well-established, particularly with respect to invasive breast cancer [142]. The initial observations were that lysyl oxidases are located in the stromal region of ductal carcinoma in situ (DCIS) breast cancer [108,109]. The highly aggressive triple negative breast cancer subtype is linked to higher LOX expression [105] (Table 2). Moreover, strong links have been found between LOXL2, metastasis [101,104,106], and higher mortality [100], and LOXL2 is a potential biomarker for cancer detection [107]. Finally in pre-clinical studies, targeting LOXL4 has been found to reduce breast cancer cell proliferation, migration, and metastasis in vitro and in vivo [102,103] (Table 2).

### 3.2. Renal Cell Cancer

Intratumoral fibrosis is a common element in clear cell renal cell carcinoma (RCC) [143], and has been suggested as a potential marker of prognosis [144]. Elevated LOX gene expression occurs in RCC [113] (Table 2) and is linked to poorer overall survival (OS) [110]. Pre-clinical studies of RCC progression showed increased LOX activity is associated with cellular adhesion, migration, and collagen stiffness [112]. LOXL2 has also been found to be significantly elevated in RCC and with poorer OS [110]. Similar to LOX, LOXL2 activity affects migration, invasion and progression to EMT in RCC [111]. 

### 3.3. Pancreatic Cancer

Pancreatic cancer (PDAC) is another tumor with well-established links to fibrosis [145] (Table 2). High expression of LOX is associated with a significantly shorter progression-free survival (PFS) [131] and poor response to chemotherapy [128,129]. The inhibition of LOX activity with a LOX-specific antibody enhanced efficacy of gemcitabine treatment in animal models [129].

### 3.4. Liver Cancer

In hepatocellular carcinoma (HCC), it is now well established that lysyl oxidase activity is significantly elevated in the tumors (Table 2) [135,136]. This elevated expression is associated with poor prognosis (OS) [134,135,136,137,138,139], higher recurrence rates [135,136], greater metastasis [137,138,139] but is also linked to immune infiltrates and T-cell activation (Table 2). Levels of LOXL2 in human sera have been suggested as a potential biomarker for HCC detection [140].

### 3.5. Lung Cancer

High expression of LOX and LOXL2 mRNA and protein is for the most part associated with poor prognosis in non-small cell lung cancer (NSCLC) patients [117,118,119,120] (Table 2). However, one histologically-based study suggests low expression of LOXL2 is associated with a poorer N-stage, higher pathological TNM stage and poorer differentiation in NSCLC patients [121]. High expression of LOXL1 in cancer-associated fibroblasts (CAFs) has been shown to enhance NSCLC tumorigenicity [33] (Table 2), while increased LOX activity also helps promote increased invasion and migration of NSCLC [123] (Table 2). 

## 4. Altered Expression of Lysyl Oxidases in Malignant Pleural Mesothelioma

Targeting fibrosis has therapeutic benefits in mesothelioma. For instance, inhibition of collagen formation by the proline analogue thiaproline delays tumor growth in a mouse model of mesothelioma [146]. More recently, the endocytic collagen receptor, uPARAP, has been identified as being a potential diagnostic marker amenable to therapeutic intervention [147]. In MPM cells derived from epithelioid and biphasic histological subtypes, invasive capacity is enhanced in fibronetin-collagen matrices [148]. Moreover, high expression of the collagen alpha type 1 (COL1A1) gene is associated with a poorer prognosis [149], while type V collagen has been linked to poorer prognosis and an altered tissue microenvironment aiding invasion [150,151].

There is a strong rationale for lysyl oxidase inhibitors as a therapeutic approach in mesothelioma due to their ant-fibrotic mechanisms [152,153], synergism with chemotherapies [1], and modulation of EMT in mesothelioma [154,155]. 

**Figure 1:**
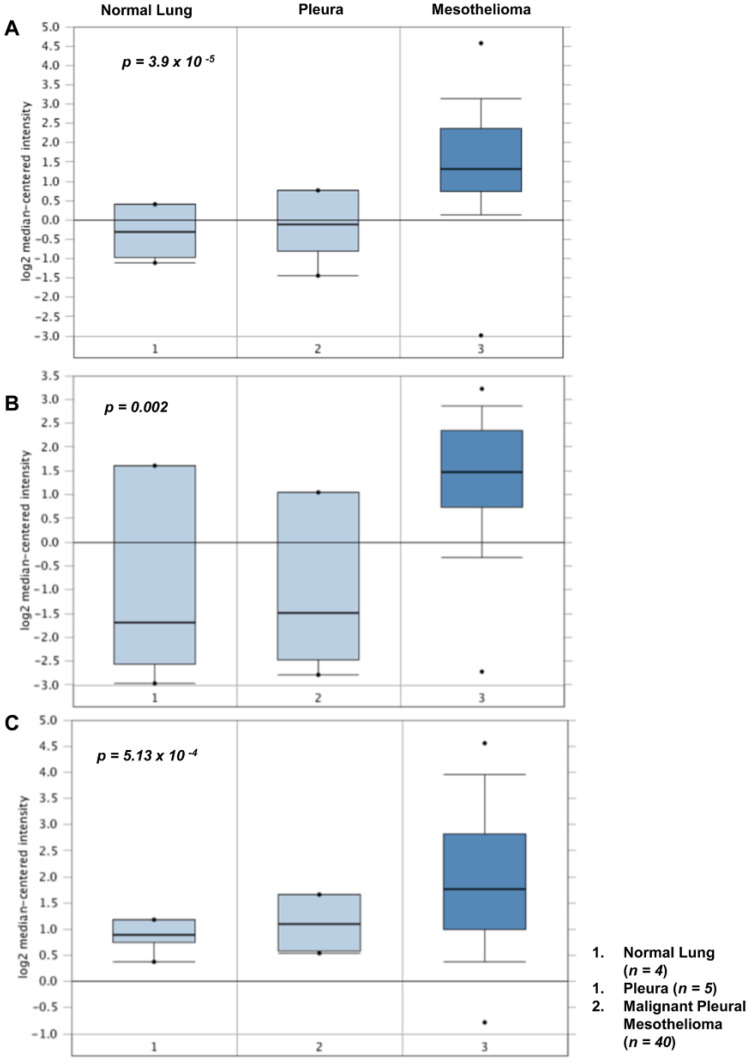
Altered expression of LOX, LOXL1 and LOXL2 in MPM. Using Oncomine [157], we found significantly altered mRNA expression of (**A**) LOX (*p* = 3.9 × 10^−5^); (**B**) LOXL1 (*p* = 0.002) and (**C**) LOXL2 (*p* = 5.3 × 10^−4^) in MPM tissues when compared to normal lung or pleura in the dataset of Gordon et al. [156].

Using Oncomine to conduct in silico analysis of existing mesothelioma microarray datasets [156,157], we confirmed that significant mRNA overexpression of lysyl oxidases occurs in both primary MPM tissues and cell lines (Figure 1), a finding since confirmed in a study of a Korean cohort of MPM patients [158].

**Figure 2:**
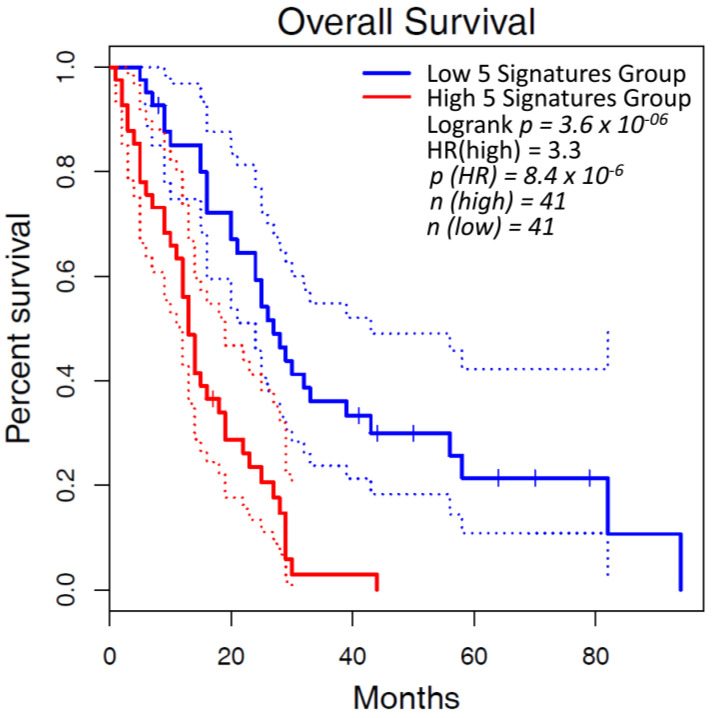
Poorer OS in mesothelioma is correlated with elevated LOX/LOXL mRNA levels. A combined gene expression of LOX, LOXL1, LOXL2, LOX3 & LOXL4 (5 signatures group) was analyzed for OS using the TCGA MESO dataset in Gepia2 [159], and stratified at the median between high expression and lox expression. When stratified at the median, the high 5 signatures group had a significantly worse OS (*p* = 3.6 × 10^−6^).

MPM datasets accessed via The Cancer Genome Atlas (TCGA) were analyzed using Gepia2 [159] to review the relationship between survivability and lysyl oxidase expression. When stratified by median expression, the results revealed that higher lysyl oxidase expression correlated with a decreased overall survival (OS) when compared to low lysyl oxidase expression (*p* = 3.6 × 10^−6^) as shown in Figure 2.

**Figure 3:**
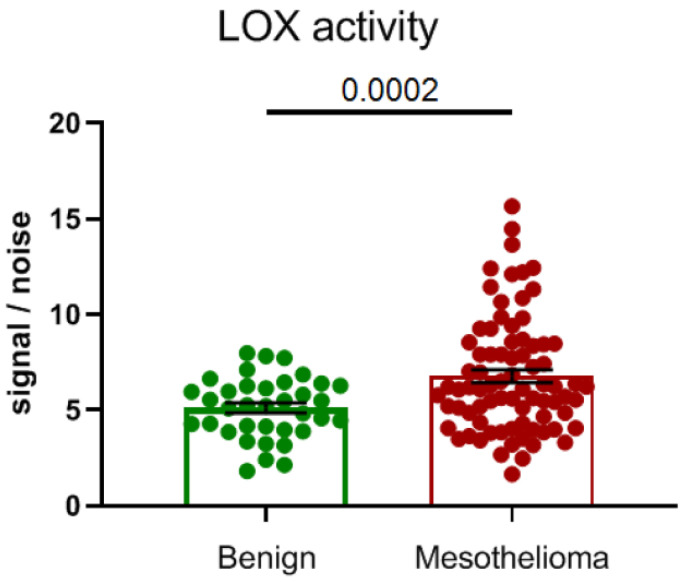
LOX activity is elevated in plasmas of patients with MPM. A cohort of *n* = 40 benign and *n* = 80 MPM plasma samples were examined for LOX activity using a LOX-specific bioprobe assay [160]. The results show a significantly elevated level of LOX activity in MPM patient plasmas (*p* = 0.0002).

Subsequently, we examined LOX activity in a cohort of *n* = 120 patient plasmas (*n* = 40 benign vs. *n* = 80 malignant) and demonstrated that LOX activity is significantly elevated (*p* = 0.0002) in the plasmas of patients with MPM (Figure 3).

Overall, the evidence suggests that LOX/LOXLs are upregulated within mesothelioma patients, play a role in the pathogenesis of MPM and are potential therapeutic targets. 

## 5. Inhibitors of Lysyl Oxidases

Many inhibitors that target the lysyl oxidase family have now been described. In the following sections, we briefly review these and their current clinical development.

### 5.1. First Generation of Lysyl Oxidase Inhibitors

#### 5.1.1. β-Aminopropionitrile (BAPN)

The first non-specific, competitive, irreversible pan-lysyl oxidase inhibitor was originally extracted from sweet peas (*Lathyrus odoratus*) in 1954 [161], with the active component being called beta-aminopropionitrile (BAPN). BAPN has a simple molecular structure containing a primary amine that mimics lysyl oxidases substrates (lysine residues), which interact with cofactor lysyl tyrosylquinone (LTQ) forming a covalent bond. In the 1960s, high doses of BAPN were trialed in Scleroderma patients (Table 3, with dosing of 1–3 g/day for 22–67 days), causing alteration in bone formations along with efficacious increases of hydroxyproline in the urine and of the dermal collagen alpha to beta ratio. A decade later, BAPN was further tested in the fibrous scar that forms in urethral stricture patients (*n* = 5), where a shorter time frame was utilized (1 g/day for 3 weeks) and no adverse effects were observed. The physical properties of the scar were affected, but insufficient data was collected to conclude a therapeutic effect [162]. BAPN is not considered to be a clinical candidate due to the potential of bone changes and off- target effects [160,163]. BAPN is also a substrate for the other amine oxidases (semi carbazide-sensitive amine oxidase (SSAO) and diamine oxidase (DAO), which may contribute to the side effects observed in clinical trials [160].

#### 5.1.2. Polyphenols Epigallocatechin Gallate (EGCG) and Ellagic Acid (EA)

Both ellagic acid (EA) and epigallocatechin gallate (EGCG) (Table 3) are dietary catechin polyphenols. Polyphenols are large molecules characterized by multiple phenyl groups that are highly oxidative and indiscriminate towards their lysine substrates. A variety of biological functions (superoxide radical scavenging activity, H_2_O_2_ scavenging, chelating capacity and lipid peroxidation) [164] have been attributed to polyphenols, potentially due to their promiscuous nature. Numerous clinical trials (Phase 1 and 2, EGCG extensively reviewed in [165] and Table 3) have been completed with EGCG and EA for both cancer and fibrosis. High doses of polyphenols are required due to poor bioavailability (EA maximal human plasma concentration 100 nM [166], and EGCG peak concentration in human plasma 341 pmol/mL [167]). In preclinical models, high doses of EGCG (2 daily doses 750 mg/kg, i.g.) are hepatotoxic to mice. Of the multiple clinical trials run on polyphenols only, one demonstrated a reduction in biomarkers (collagen oligomeric matrix protein and periostin) and a reduction in protein expression in treated tissues (col1, snail and pSMAD, NCT03928847) [46]. The EGCG-dependent reduction in fibrotic markers is a promising result, but to date no therapeutic effects or clinical benefits have been demonstrated. Polyphenols’ influence on multiple biological processes, poor bioavailability and potential off-target effects will need to be addressed before they can become a successful clinical candidate. 

#### 5.1.3. Copper Chelators (Tetrathimolybdate and D-Penicillamine)

Copper is an essential microelement involved in a plethora of biological processes. Unbound copper ions are a potent oxidant and serve as a cofactor in redox reactions for enzymes, including lysyl oxidases. Copper absorption and its homeostasis in the body is highly regulated and complex, particularly as an excess is cytotoxic. The concentration of free copper in human plasma ranges from 66–143 μg/mL and in a variety of cancers has been shown to be elevated to between 129–328 μg/mL [168]. Copper staining of cancer tissue indicate that fibrosis is increased [169]. Copper concentration is particularly relevant to lysyl oxidases, as sequestering of copper is crucial for catalytic activity and the protein folding of the LTQ complex. A full review of copper chelators is not within the scope of this article; please see Baldari et al. [170]. 

The most commonly used copper chelators in clinical trials are tetra-thimolybdate (TM) and D-penicillamine, (Table 3), which are known to be well-tolerated at low doses. The only clinical trial so far that demonstrated a reduction in fibrosis had 80% of patients withdraw from the trial due to adverse effects. Despite exceptionally high doses (D-penicillamin: 750–1000 mg/mL daily) utilized, most fibrotic tissues were unchanged, with the exception of a decrease in cardiomegaly. To advance copper chelators in clinical trials, the dose-dependent toxicity needs to be addressed.

**Table 3:** Inhibitors of the lysyl oxidase family and their current clinical development.

Inhibitor	Company	Molecule Type	Target	Clinical Details	Clinical Trial Identifier and Name	Disease	Summary Results	Reference
Beta-aminopro pionitrile (BAPN)	National heart Institute	Small molecule inhibitor	Pan-LOXs, SSAO and DAO	Phase 1–3 g/day for 22–67 days *n* = 4		Scleroderma	↑urine HYD ↑α:β collagen chains implying ↓crosslinks Bone formation changes	[171]
Beta-aminopro pionitrile (BAPN)	University of Arizona	Small molecule inhibitor		250 mg 4 times daily for 3 weeks *n* = 5		Fibrous based urethral strictures	No adverse effects. Unclear therapeutic benefit. Demonstrated efficacy. ↑ in acid-soluble collagen. ↓ dermal scar strength.	[162]
Simtuzumab (GS-6624)	Gilead	Humanised Antibody	LOXL2	Simtuzumab (200 or 700 mg) with Ruxolitinib 6 cycles of 28 days (~6 months) *n* = 54	Phase 2 NCT01369498	Thrombocythaemia myelofibrosis	No clinical benefit in bone marrow fibrosis	[172]
Simtuzumab (GS-6624)	Gilead	Humanised Antibody	LOXL2	75 mg or 125 mg subcutaneous for 96 weeks *n* = 234	Phase 2 NCT01672853	Liver fibrosis in adults with primary sclerosing cholangitis	No significant clinical benefit to patients	[173]
Simtuzumab (GS-6624)	Gilead	Humanised Antibody	LOXL2	Subcutaneous 125 mg/mL single dose once a week. Up to 254 wks	Phase 2 NCT01769196, NCT01759511	Idiopathic pulmonary fibrosis	No ↑ progression free survival. Not recommended to progress in IPF	[174]
Simtuzumab (GS-6624)	Gilead	Humanised Antibody	LOXL2	Intravenous 700 mg every 2 wks for 22 wks (~6 months)	Phase 2 NCT01707472	Chronic Liver fibrosis in HIV and HCV–infected adults	Well tolerated and modulation of TGFB3	[175]
Simtuzumab (GS-6624)	Gilead	Humanised Antibody	LOXL2	Combined with Gemcitabine (1000 mg/m^2^) Simtuzumab either 200 or 700 mg (~3 months of treatment) *n* = 240	Phase 2 NCT01472198	Metastatic pancreatic adenocarcinoma	No ↑ OS (overall survival)	[176]
Simtuzumab (GS-6624)	Gilead	Humanised Antibody	LOXL2	Combination with FOLFIRI, Simtuzumab 200 or 700 mg *n* = 249 (second line) (~6 months of treatment)	Phase 2 NCT01479465	Metastatic KRAS mutant colorectal adenocarcinoma	Simtuzumab did not improve clinical outcomes	[177]
Simtuzumab (GS-6624)	Gilead	Humanised Antibody	LOXL2	Subcutaneous weekly injections of 75 or 125 mg of Simtuzumab over 240 wks *n* = 219	Phase 2 NCT01672866; NCT01672879;	Liver Fibrosis (nonalcoholic steatohepatitis, NASH)	Simtuzumab did not improve clinical outcomes	[178]
Simtuzumab (GS-6624)	Gilead	Humanised Antibody	LOXL2	200 mg, 700 mg or placebo by intravenous infusion every 2 weeks *n* = 258	Phase 2 (NCT01672879)	Nonalcoholic Steatohepatitis	Regression of fibrosis associated reduced liver-related complications	[179]
Epigallocatechin Gallate (EGCG)	Northumbria University	Polyphenol	Aldehydes	Oral 135 and 270 mg single dose	Phase 1 NCT00981292	Healthy subjects	No adverse effects	No Results Posted
Epigallocatechin Gallate (EGCG)	The University of Texas Health Science Center at San Antonio	Polyphenol	Aldehydes	Oral 450 mg twice a day for 1 year *n* = 50	Phase 1 NCT02891538	Primary colon or rectal adenocarcinoma	Study completion in 2023	No Results Posted
Epigallocatechin Gallate (EGCG)	National Cancer Institute (NIH)	Polyphenol	Aldehydes	Oral 800 or 1200 mg for 14–28 days prior to resection	Phase 2	Bladder cancer	PK: EGG levels increased. No significant changes in biomarkers	[180]
Epigallocatechin Gallate (EGCG)	University of California, San Francisco	Polyphenol	Aldehydes	Patients: 600 mg EGCG capsules once daily by mouth for two weeks	Phase1 NCT03928847	Idiopathic pulmonary fibrosis	Reduction in serum biomarkers collagen oligomeric matrix protein (COMP) and periostin and in tissue Col1, snail, pSMAD3, fibronectin; *n* = 4	[181]
Tetrathiomolybdate (TM)	University of Michigan	Copper chelator	Copper	*n* = 23	Phase1/2 NCT00189176	Idiopathic pulmonary fibrosis	Completed in 2006	No Results Posted
Tetrathiomolybdate (TM)	New York University School of Medicine & University of Michigan	Copper chelator	Copper	*n* = 30	Phase 2	Malignant Pleural Mesothelioma	Following cytoreduction surgery, antiangiogenic effects observed with minimal toxicity.	[182]
Tetrathiomolybdate (TM)	University of Michigan	Copper chelator	Copper	Oral 180 mg/day *n* = 15	Phase 2	Advanced kidney cancer	Well-tolerated and reduces copper in serum. Potential as an antiangiogenic therapy	[183]
Tetrathiomolybdate (TM)	University of Michigan	Copper chelator	Copper	*n* = 18 90, 105, 120 mg/day 90 days	Phase 1	Metastatic solid tumors including breast, colon, lung, and prostate cancers	Toxicity: mild anemia	[184]
Tetrathiomolybdate (TM)	Weill Cornell Medicine Iris Cantor Breast Center	Copper Chelator	Pan-LOXs	Oral 8–17 mg/dL for 2 years *n* = 75	Phase 2 NCT00195091	Breast Cancer stage II triple-negative breast cancer (TNBC), stage III and stage IV without any evidence of disease (NED)	No significant ↑ OS	[185]
ATN-224	Cancer research UK	Copper chelator	Copper	Once daily	Phase 2 NCT00674557	Breast cancer	Terminated 2009 No results posted	No Results Posted
D-penicillamine	National institute of respiratory diseases in Mexico City	Copper Chelator	Non specific Pan-LOXs	Daily 600 mg *n* = 56 Combined with colchicine 1 mg daily and prednisone 15 mg/d 5 year study	Phase 2	Idiopathic pulmonary fibrosis	No improvement in disease progression	[186]
D-penicillamine	university of California	Copper Chelator	Non specific Pan-LOXs	Oral 750–1000 mg/day or 125 mg *n* = 134 24 months study	Phase 2	Diffuse cutaneous systemic sclerosis	High dose had 80% adverse event related withdrawal A reduction in cardiomegaly	[187]
D-penicillamine	New approaches to brain tumour therapy CNS consortium (NCI)	Copper Chelator	Non specific Pan-LOXs	250 mg/day *n* = 40	Phase 2	Glioblastoma —post resection	Adverse effects: hypocupremia No change in survival	[188]
PXS-5505	Pharmaxis	Small molecule inhibitor	Pan-LOXs	Orally as 2 × 100 mg twice a day	Phase 1/2a NCT04676529	Myelofibrosis	NA	NA
PXS-5505	university of Rochester	Small molecule inhibitor	Pan-LOXs	Orally 100–200 mg BID in combination with Atezolizumab (Anti-PD-L1) 1200 mg every 3 weeks and Bevacizumab (Anti-VEGF) 15 mg/kg every 3 weeks	Phase 1b/2 NCT05109052	Unresectable hepatocellular Carcinoma	NA	NA
PXS-6302	Pharmaxis	Small molecule inhibitor	Pan-LOXs	Escalating dose 0.6–8 mg for 7 days topical	Phase 1/1c SOLARIA I ACTRN12621000322831	Healthy subjects Acute and established scar	NA	NA
PXS-5382	Pharmaxis	Small molecule inhibitor	LOXL2/3	Single dose	Phase 1 NCT04183517	Healthy subjects	No adverse effects	No Results Posted
PXS-5338	Pharmaxis	Small molecule inhibitor	LOXL2/3	Single dose	Phase 1 ACTRN12617001444370	Healthy subjects	No adverse effects	No Results Posted [160]
PAT-1251 (GB2064)	PharmAkea (Now with Galecto)	Small molecule inhibitor	LOXL2	Oral 150–4000 mg single dose	Phase 1 NCT02852551	NA	No adverse effects	NCT02852551
PAT-1251 (GB2064)	PharmAkea (Now with Galecto)	Small molecule inhibitor	LOXL2	Oral 150–4000 mg single dose orally as 4 × 250 mg tablets twice a day	Phase 2a MYLOX-1 NCT04679870 NCT04054245—withdrawn MD Anderson	Myelofibrosis.	NA	NCT04679870

### 5.2. 2nd Generation Inhibitors

#### Lysyl Oxidase like 2 Antibodies (Simtuzumab) 

Simtuzumab was the first humanized monoclonal antibody to target the enzymatic activity of LOXL2 to enter the clinic. Expectations were high for Simtuzumab, as it was developed as other anti-fibrotic treatments (nintedanib and pirfenidone) were being approved by the FDA for IPF. In addition, there was a prevalence of promising preclinical data (utilizing the mouse LOXL2 antibody AB0023) in both cancers and organ fibrosis [189]. However, in the clinic, despite Simtuzumab being well-tolerated, it did not show efficacy in both cancers and fibrotic diseases (Table 3). The lack of efficacy of Simtuzumab is thought to be dependent on a number of factors—which are irrespective of the potential of targeting the lysyl oxidase family in cancer and fibrotic diseases [160]. 

The inability to measure lysyl oxidase activity in vivo during Simtuzumab clinical trials limits our understanding of target engagement, pharmacokinetics (including determining optimal dosing, tissue distribution and process of elimination) and pharmacodynamics, which limits the ability to optimize the clinical trials. This lack of understanding is potentially the reason why the early trials (in primary sclerosing cholangitis, IPF and NASH) were dosed with only 125 mg, which, based on preclinical studies (Table 3), was insufficient for significant target inhibition. Later cancer trials dosed patients with 700 mg for 3–6 month periods; however, these trials recruited patients irrespective of LOXL2 expression, activity or presence of fibrosis, which may have diluted efficacious responses to Simtuzumab. 

Simtuzumab targets a single member of the lysyl oxidase family (LOXL2). To date, it is unclear if there is redundancy of the lysyl oxidases expression when a single member is targeted in humans. In a cancer setting, redundancy alone could explain the absence of efficacy within the clinical trials. Furthermore, it is questionable if large molecules like Simtuzumab are able to penetrate the complex impervious environment of fibrosis, which is pivotal for the success of human clinical trials in either cancer or fibrosis. 

The failure of Simtuzumab was disappointing, but may be explained by the unavailability of target engagement assays leading to insufficient dosing, lack of understanding of protein turnover, lysyl oxidase family redundancy and the potential for poor tissue penetration. Despite this, Simtuzumab blazes the path for the future development of small-molecule approaches and target engagement-driven clinical trials for the lysyl oxidase inhibitors. 

### 5.3. Lysyl Oxidase Small Molecule Inhibitors: The Way of the Future

The first generations of lysyl oxidase inhibitors have shaped the numerous clinical trials that are currently underway in cancer and fibrosis. For example, all current trials are wielding oral small-molecule enzymatic inhibitors, which should be optimal for tissue penetration (Table 3). Furthermore, target engagement assays have been developed utilizing Quanterix technology to measure low concentrations and activity of LOX and LOXL2 specifically, in both tissue and plasma [160]. The plasma LOX and LOXL2 activity has been shown to mirror recovery of activity in human tissue, providing a less invasive monitoring of target engagement. The establishment of target engagement assays has illustrated the dynamic nature of protein resynthesis, particularly for LOXL2 [160]. Despite reaching 90% target engagement, daily treatments are required to ensure complete inhibition due to the high resynthesis rates of lysyl oxidases [160]. Presently, in terms of treatment, it is still unclear the role redundancy will play within the lysyl oxidase family, but this will only be a consideration when not utilizing pan-lysyl oxidase inhibitors (e.g., PAT-1251 a LOXL2 inhibitor and PXS-5382 a dual LOXL2/LOXL3 inhibitor). Redundancy is more likely to occur in a cancer setting due to the plasticity and adaptive nature of tumors; therefore, a pan-lysyl oxidase inhibitor is generally a better approach. The new generation lysyl oxidase inhibitors have been shown to be non-toxic in human subjects in Phase 1 dose escalation trials (PXS-5505, PXS-6302, PXS-5382, PXS-5338 and PAT-1251, Table 3). Phase 1c/2 recruitment for clinical trials for Myelofibrosis has opened for both the pan-lysyl oxidase inhibitor (PXS-5505) and a LOXL2 specific inhibitor (PAT-1251) (Table 3). A combination clinical trial (PXS-5505 and Atezolizumab (anti-PD-L1) and Bevacizumabin (anti-VEGF) in hepatocarcinoma will begin patient recruitment imminently. For skin fibrosis, a topical pan-lysyl oxidase inhibitor PXS-6302, will soon open to recruit patients with acute and established scars. 

## 6. Discussion

Given the significant roles of lysyl oxidases in both cancerous and fibrotic disease settings, coupled with the current development of small molecule inhibitors, it is our opinion that a strong rationale has emerged for the progression to clinical trials in mesothelioma. In the previous sections, we have documented the significant roles played by lysyl oxidases in fibrosis not only in the non-cancerous, but also in the cancerous setting. We have presented evidence that lysyl oxidase expression and activity are significantly dysregulated in mesothelioma. Lysyl oxidase inhibitors have been predominantly tested as a single agent, where it is well-established that their primary mechanism of action is to prevent enzymatic cross-linking of collagen and elastins to counter fibrosis. The anti-fibrotic action of lysyl oxidase inhibitors has been demonstrated in a variety of preclinical cancer [125,190] and fibrosis models [189]. In cancer, lysyl oxidase inhibitors alone are not cytotoxic, but are thought to be ideal for combining with chemotherapies and/or immune therapies.

Chemotherapies have been shown to increase the tissue stiffness and fibrosis in tumors [125,190]. Desmoplastic pancreatic cancers are well-circumscribed by fibrotic tissue, increasing interstitial pressure and limiting immune infiltration and the penetration of chemotherapies. Lysyl inhibitors have been shown to soften tumors and have a synergistic effect with chemotherapy (Gemcitabine) to increase survival and decrease metastasis [125]. Chemotherapies are beneficial in mesothelioma patients, although they may also exacerbate lung fibrosis, further limiting their respiration and lymphatic drainage. It is anticipated that lysyl oxidase inhibitors could alleviate mesothelioma patients’ fibrotic response to chemotherapies, increase immune infiltration, extend survival and increase the quality of life. 

The synergistic effects of chemotherapies and lysyl oxidase inhibitors have previously been thought to be solely due to the anti-fibrotic effects. However, more recently, lysyl oxidase inhibition has impacted immune cells. Chen et al. 2019 first suggested that lysyl oxidase inhibitors prevent the chemoattraction of tumor-associated macrophages (TAMs, pro-tumorigenic) in preclinical models of glioblastoma [191]. Furthermore, in pancreatic tumors treated with both a lysyl oxidase inhibitor and immune therapy, a decrease in pro-tumorigenic TAMs and an increase in CD8+ Granzyme B+ T-cells (anti-tumorigenic) was associated with increased survival [125]. 

Within the setting of metastasis, it may be possible to use lysyl oxidase inhibitors to prevent metastasis. Responses to paclitaxel-based therapy in breast cancer models induced LOX levels and activity in the lungs, resulting in pulmonary ECM remodeling and a pro-metastatic niche [192]. Mechanistically, CD8+ T cells secreted LOX, which could be targeted with lysyl oxidase inhibitors to suppress metastasis [192]. 

In preclinical models of human hepatocellular carcinoma, high LOXL4 expressing macrophages have an immunosuppressive function via an interferon (IFN)-mediated signal transducer and activator of the transcription (STATs)-dependent PD-L1 activation on CD8+ T cells [193]. Disappointingly, they fail to demonstrate that lysyl oxidase inhibitors affect survival and the immunosuppressive environment. Despite this, a phase 1c/2a trial combining a lysyl oxidase pan inhibitor with standard of care (Atezolizumab plus Bevacizumab) in unresectable hepatocellular carcinoma will be underway in 2022. 

Checkpoint inhibitors have been approved by the FDA for the treatment of mesothelioma [11]. Coupled with lysyl oxidase inhibitors’ anti-fibrotic activity and possible regulation of immune cell infiltration [125,194], their combination has potential as a treatment option for mesothelioma. Moreover, anti-fibrotic strategies could be combined with standard first-line chemotherapies (cisplatin/pemetrexed) and conceivably be added to second-line salvage settings. However, significant preclinical studies will be required to test these promising combination therapies.

Additional remaining unanswered questions that may affect the inclusion of anti-fibrotic treatment strategies in mesothelioma include:

1. Is fibrosis limiting lymphatic drainage to cause pleural fluid buildup? Can lysyl oxidases inhibitors alleviate pleural fluid buildup?
2. Will lysyl oxidase inhibitors be synergistic with chemotherapies and immune therapies in patients?
3. How do lysyl oxidase inhibitors affect macrophages and their interaction with T-cells?
4. Will tumors adapt to lysyl oxidase inhibition?
5. Is there redundancy in the lysyl oxidase family members, particularly when treated with lysyl oxidase inhibitors?
6. Are LOX/LOXL inhibitors synergistic with immune therapies and/or chemotherapies in preclinical mesothelioma and patients?

In this review, we elaborated on the known functions of lysyl oxidases in the fibrotic setting. Overall, we believe that lysyl oxidases play important roles in the pathogenesis of mesothelioma. The current developments regarding lysyl oxidase inhibitors in the clinical setting are very encouraging and it will be exciting to see how these clinical trials unfold. Targeting lysyl oxidases has potential therapeutic implications in the management of mesothelioma.

## 7. Conclusions

One of the most significant issues facing patients with mesothelioma is a drastically reduced quality of life. A debilitating symptom of mesothelioma is dyspnea, and it is our belief that fibrosis plays a major role. Targeting lysyl oxidases to disrupt the fibrotic process may improve the mesothelioma patients’ quality of life not only whilst undergoing therapy, but also in a palliative setting.

A second critical aspect of targeting lysyl oxidases is the potential for the anti-fibrotic effects to either potentiate or improve the response of patients to immune checkpoint inhibition or in combination with standard chemotherapy. Drugs that target lysyl oxidases could be combined into all existing therapeutic regimens for the treatment of mesothelioma to the potential benefit of patients.

## Figures and Tables

**Table 1:** Selected examples of known LOX/LOXL family links with various non-cancerous settings.

Idiopathic Pulmonary Fibrosis (IPF)	
β-aminopropionitrile (BAPN) found to inhibit pulmonary fibrosis in a lung model of silicosis	[34]
LOX activity induced in bleomycin-induced lung fibrosis and alleviated by treatment with LOX/LOXL inhibitor	[35,36,37]
In IPF patients, elevated serum levels of LOXL2 are associated with an increased risk for disease progression.	[38]
LOX activity promotes the progress of EMT in a paraquat model of IPF.	[39,40]
A comparative analysis shows that LOX/LOXL2 are elevated in IPF fibroblasts, while LOXL2/3 activity is crucial for fibroblast-to-myofibroblast transition (FMT).	[41]
Elevated serum levels of LOXL2 are associated with rheumatoid arthritis -associated interstitial lung disease (RA-ILD).	[42]
Increased collagen fibril thickness in IPF versus non-IPF lung tissues is correlated with increased levels of LOXL1/LOXL2 protein, and a decrease in LOX protein expression.	[43]
Loss of LOXL1 activity prevents the development of fibrosis in a transforming growth factor-β1-induced model of pulmonary fibrosis.	[44]
In bleomycin-induced lung fibrosis, nuclear expression of LOXL2 appears to be a major element in the progression of lung fibrosis.	[45]
LOXL2 inhibitor induces collagen turnover in ex vivo lung explants from patients with IPF.	[46]
In a study comparing extracellular vesicles (EVs) from IPF-derived pulmonary fibroblast cell lines versus normal pulmonary fibroblast cell lines for differentially expressed proteins, LOXL1 was found in IPF EVs.	[47]
Kidney Fibrosis	
LOXL2 is expressed in compartments of renal tissue, where it appears to contribute to the progression of tubulointerstitial fibrosis.	[48]
LOXL2 inhibition significantly reduced interstitial fibrosis in a mouse model of renal fibrosis.	[49]
Elevated serum LOX and LOXL2 levels may act as a potential biomarker for kidney fibrosis.	[50]
In a murine model of cyclosporine induced nephropathy, pan-LOX and LOXL2 specific inhibitors attenuated kidney damage.	[51]
Liver Fibrosis	
Increased LOX levels both in tissue and serum is associated with collagen in the extracellular space in animal models of hepatic fibrosis.	[52,53,54,55]
Hepatic stellate cell activation results in elevated LOX mRNA and protein in liver fibrosis.	[56,57,58,59]
Lysyl oxidase activity levels increase in patient serum from chronic persistent hepatitis to chronic active hepatitis to cirrhosis.	[60]
In a mouse model of liver fibrosis, increased steady state levels of LOXL mRNA occur early in fibrosis development.	[61]
Expression of LOX and LOXL2 in hepatocytes is linked to liver fibrosis.	[62]
Reduced levels of miR-29b are associated with elevated levels of LOX in models of liver fibrosis.	[63]
In patients with severe obesity or obstructive sleep apnoea, serum levels of lysyl oxidase can act as a potential biomarker of liver fibrosis.	[64]
Targeting of LOXL2 is associated with anti-fibrotic effects in a mouse model of hepatic fibrosis.	[65]
Links between LOXL2, insulin resistance and fibrosis accumulation in non-alcoholic fatty liver disease (NAFLD) are identified.	[66]
LOXL1 identified as a candidate therapeutic target for ameliorating liver fibrosis progression in cirrhosis, and inhibition of human hepatic stellate cell mediated fibrogenesis.	[67,68]
First demonstration that a small molecule dual inhibitor of LOXL2/3 (PXS-5153A) can ameliorate fibrosis in models of liver fibrosis and myocardial infarct	[69]
Patients with HCV who demonstrated sustained responses to antiviral therapy were shown to have regression in liver fibrosis associated with decreased LOXL2 expression.	[70]
anti-LOXL2 based therapy in a mouse model of liver fibrosis results in reduced fibrosis via accelerated collagenolytic activity by macrophages.	[71]
miR-15b/16 are downregulated in activated hepatic stellate cells (HSCs), and overexpression of these miRs is found to suppress LOXL1 expression in HSCs and induce a fibrogenic response.	[72,73]
Selective deletion of LOXL1 in HSCs in a murine NAFLD model ameliorates fibrosis, and serum levels of LOXL1 are positively correlated with histological fibrosis progression in NAFLD patients.	[74]
Amyotrophic Lateral Sclerosis (ALS)	
LOX transcripts are overexpressed in patient lumbar spinal cord samples.	[75]
LOX activity increased in animal model of ALS.	[76]
Systemic Sclerosis	
LOX mRNA transcripts overexpressed in fibroblasts from patients with systemic sclerosis	[77,78]
Elevated levels of LOX found in the serums of patients with systemic sclerosis	[78,79]
Elevated levels of LOX and LOXL2 in skin and lungs of systemic sclerosis patients	[37]
Elevated serum LOX levels and idiopathic pulmonary arterial hypertension (iPAH) found in patients with systemic sclerosis	[80]
LOXL4 activity as a cause of cutaneous fibrosis in fibroblasts from patients with system sclerosis identified	[81]

**Table 2:** Selected examples of known LOX/LOXL family links with various cancers.

Breast Cancer	
Lysyl oxidases associated with chemotherapy resistance in triple negative breast cancer	[99]
Stromal expression of LOXL2 is associated with tumor aggression and disease-specific mortality.	[100]
Oncostatin-M-induced ECM remodelling via upregulated LOXL2	[101]
Inhibition of LOXL4 decreased breast cancer cell proliferation, migration, and metastasis in vitro and in vivo.	[102,103]
LOXL2 promotes tumour lymph-angiogenesis and lymph node metastasis.	[104]
LOX expression is significantly higher in triple negative breast cancers versus other breast cancer subtypes.	[105]
LOXL2 involved with breast cancer metastasis to the lung	[106]
LOXL2 expression may serve as a biomarker for breast cancer and is detectable in serum and urine.	[107]
LOX and LOXL proteins are located in the stromal reaction of ductal carcinoma in situ (DCIS) breast cancer.	[108,109]
Renal Cell Cancer (RCC)	
LOX and LOXL2 significantly elevated in RCC and associated with poorer overall survival (OS)	[110]
LOXL2 associated with migration, invasion and EMT transition of RCC	[111]
Primary RCC cultuure endogenously express LOX, and plays major roles in progression via activities on cellular adhesion, migration, and collagen stiffness.	[112]
Early demonstration of overexpression of LOX mRNA in RCC	[113]
Non-Small Cell Lung Cancer (NSCLC)	
A novel LOX polymorphism G473A is associated with increased risk for lung cancer.	[114,115,116]
LOXL1 promotes lung cancer tumourigenicity via collagen matrix remodelling and collagen fibre alignment in vitro and in vivo.	[33]
High expression of LOX and LOXL2 mRNA and protein is associated with poor prognosis in NSCLC patients.	[117,118,119,120]
Low expression of the LOXL2 protein in adenocarcinomas is associated with a poorer N-stage, a higher pathological TNM stage and poorer differentiation.	[121]
The miR-200/ZEB1 axis drives lung cancer metastasis through LOXL2.	[122]
LOX activity is associated with increased invasion and migration of hypoxic NSCLC cells.	[123]
Reduced levels of microRNA-29a (miR-29a) in lung cancer is associated with overexpression of LOXL2 and concomitant fibrosis.	[124]
Pancreatic Cancer (PDAC)	
Tumor stiffening reversion through collagen crosslinking inhibition improves T-cell migration and anti-PD-1 treatment.	[125]
LOXL2 is highly up-regulated (≥20-fold) in the PDAC secretome.	[126]
High expression of LOXL2 protein is associated with worse DFS and OS in patients with PDAC.	[127]
Increased levels of LOX, LOXL1, and LOXL2 expression in PDAC are associated with poor responses to chemotherapy by limiting drug distribution, and inhibition of LOX enhances drug efficacy.	[128,129]
In an orthotopic PDX model of PDAC, targeting LOXL2 led to accelerated tumour growth, and poorer overall survival.	[130]
Levels of LOX are a potential prognostic markers for the prognosis of pancreatic cancer patients.	[131]
Liver Cancer	
Reduced levels of miR-29a in hepatocellular carcinoma (HCC) lead to elevated expression of its known targets LOX and LOXL2.	[132]
A circRNA network has been identified that activates LOX transcription in HCC.	[133]
High LOXL3 expression predicts poor outcomes for patients with HCC, and is correlated with immune infiltrates and T-cell activation.	[134]
High LOX expression is associated with an higher recurrence rate and poorer OS in patients with HCC.	[135,136]
LOXL2 is overexpressed in HCC and positively correlated with tumour grade, metastasis, and poor OS.	[137,138,139]
LOXL2 is significantly overexpressed in human HCC sera and may act as a good biomarker for HCC.	[140]
LOXL4 is upregulated in HCC tissues and associated with poor prognosis. Exosomal-mediated transfer of LOXL4 between HCC cells and human umbilical vein endothelial cells (HUVECs) promotes cell migration and angiogenesis, respectively	[141]

## Data Availability

The datasets analyzed during the current study are available in the following repositories: Oncomine: https://www.oncomine.org/resource/login.html (accessed on 26 March 2019); and Gepia2: http://gepia2.cancer-pku.cn/#index (accessed on 17 January 2022).
